# A Common Denominator: Calculating Hospitalization Rates for Ambulatory Care–Sensitive Conditions in California

**Published:** 2011-08-15

**Authors:** Camillia Lui, Steven P Wallace

**Affiliations:** University of California, Los Angeles (UCLA); UCLA School of Public Health and UCLA Center for Health Policy Research, Los Angeles, California

## Abstract

**Introduction:**

Chronic health conditions are considered ambulatory care–sensitive conditions (ACSC) when the illness is controllable with effective and timely outpatient care that can potentially prevent the need for hospitalizations. Hospitalization rates for ACSC serve as an indicator of the access to and quality of primary care for chronic conditions. Standard methods to calculate hospitalization rates incorporate the total population in the denominator instead of the total population at risk for a hospitalization. By accounting for people with an ACSC, this study compares standard methods to a disease prevalence–adjusted method to highlight the importance of adjusting for ACSC prevalence when using ACSC hospitalizations in assessing primary care outpatient services.

**Methods:**

We combined California Health Interview Survey and hospital discharge data to calculate standard (crude and age-adjusted) and disease prevalence-adjusted hospitalization rates for hypertension and congestive heart failure. To compare rate calculations, we ranked California counties by their hospitalization rate.

**Results:**

Counties had high prevalence and low numbers of hospitalizations for hypertension; their rankings for hospitalization rates for hypertension did not vary, even after accounting for prevalence. In contrast, counties had low prevalence and high numbers of hospitalizations for congestive heart failure; their rankings varied substantially for congestive heart failure after accounting for prevalence.

**Conclusion:**

Because the number of people diagnosed with an ACSC is rising and costs to treat these conditions are increasing, our findings suggest that more accurate measures of ACSC hospitalization rates are needed. Incorporating disease prevalence will contribute to ACSC research by improving the validity of hospitalization rates as a measure for quality of primary care services.

**Figure:**
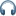
Listen to an interview with Camillia Lui, winner of the inaugural Preventing Chronic Disease Student Paper Competition. (MP3 1.07Mb)

FRAN KRITZ I’m Fran Kritz for *Preventing Chronic Disease*. We’re speaking by phone with Camillia Lui, a graduate student at the UCLA School of Public Health. Camillia Lui is the winner of the inaugural *Preventing Chronic Disease* Student Paper Competition. Camillia, tell us about your paper. What was the title and what were your findings?
CAMILLIA LUI This is quite an honor to get this award, so I’m very appreciative to the journal. The title of the article is “A Common Denominator: Calculating Hospitalization Rates for Ambulatory Care–Sensitive Conditions in California.” I had the opportunity to work with Steven Wallace at the UCLA Center for Health Policy Research on this — on this paper. It was actually a grant received from the California HealthCare Foundation to examine the burden of chronic diseases in California. So, 1 of the ways to examine the burden of chronic disease is to use preventable hospitalization rates. If an area has good access to quality primary care services, the idea is that then there are lower hospitalization rates. So, we’re using that as our key variable for this paper.
FRAN KRITZ And what — tell us the methodology that you used to study the target areas.
CAMILLIA LUI So, we decided to use 2 ways of calculating the hospitalization rates, the traditional way of crude and age-adjusting versus a disease-prevalence method. And the traditional way [is you] usually use the total population in the county as the denominator to calculate the rate. But not everyone is at risk for a hospitalization, especially those who have been diagnosed with a chronic condition. So, we’re fortunate enough to merge in data from the California Health Interview Survey to this data to capture the disease prevalence. So, to capture those who have been diagnosed with a chronic condition, so we used that as the denominator for calculating the rates. And, for this paper, we actually compared the rates using 2 chronic conditions that are manageable with outpatient care, and that was hypertension and congestive heart failure.
FRAN KRITZ And what is the significance of your findings? How do they relate to community care?
CAMILLIA LUI Well, it’s kind of interesting because when we looked at the hypertension rates, it doesn’t matter which method we used. There were very few differences when we compared it by county rankings, but when we were looking at the congestive heart failure, there were actually larger differences with the county rankings such that there are certain counties that just showed a higher burden, using the prevalence-adjusted rate, that wouldn’t have shown that when we looked at the traditional age-adjusted rate. So, it doesn’t mean that 1 way is better than the other, but it does highlight that there might be some underlying differences that traditional methods for calculating hospitalization rates — that they won’t capture.
FRAN KRITZ What was it like working with a mentor?
CAMILLIA LUI It was great. Dr Wallace is definitely for the students, and he gave me the guidance that I needed, but also gave me the sort of freedom to pursue it. We originally did this project for the California HealthCare Foundation, but the next step was to publish it, and through this opportunity, the student contest, we were able to at least submit a manuscript, for it.
FRAN KRITZ And, what’s next for you? What will you be studying?
CAMILLIA LUI So, 1 of the next steps is we are actually going to take these rates and see if they have different factors that are associated with it — associated with, like, access to care factors. The other thing that was really interesting about this project is that the Healthcare Foundation — California HealthCare Foundation, they took the data and were able to make it into this very user friendly, interactive website. So, folks from the county level to public health researchers, to policy makers, they can actually go to this website and click on this — the California county and see what the — what the data look like for chronic disease for their area. So, that was 1 thing that I really enjoyed with this, being able to take data, information about chronic disease and make it readily available and user friendly.
FRAN KRITZ What do you see you doing down the road with your career in public health in addition to the really fine research you’re doing right now?
CAMILLIA LUI I’m definitely a research nerd. One of the things that drove me back to school is to gain the quantitative skills, but to be able to take that back to the community and especially work with community-based organizations with that. There’s just so much data out there, whether it’s community organizations collecting it for themselves or using data such as California Health Interview Survey data to better inform their own work, documents, problems, and strengths of their communities, and inform them for better programs and implementation and service delivery.
FRAN KRITZ Sounds exciting. Camillia Lui, winner of the inaugural *Preventing Chronic Disease* Student Paper Competition, congratulations. I have no doubt we’re hearing — we’ll be hearing lots, both about your research and other activities in the field of public health in the future. Thank you so much.
CAMILLIA LUI Thank you, Fran.
FRAN KRITZ I’m Fran Kritz for *Preventing Chronic Disease*.

## Introduction

Chronic health conditions are the leading cause of death and disability in the United States and are the largest component of health care costs (1,2). Some chronic health conditions are deemed ambulatory care–sensitive conditions (ACSC) because they are controllable with effective and timely outpatient care and disease management (3). Rates of ACSC hospitalizations are used by public health officials to measure accessibility and effectiveness of primary health care services (4-6) and to control health costs for ACSCs. The Agency for Healthcare Research and Quality (AHRQ) used hospital inpatient discharge data to develop a set of Prevention Quality Indicators (PQIs) to assess ACSC hospitalizations (7,8).

Common ways to report hospitalization rates include crude and age-adjusted rates (9,10). These rate calculations use the total number of people in a population. Crude rates often serve as a summary measure that captures the overall burden of hospitalizations in the total population (11). ACSCs may be confounded by individual demographic characteristics, such as age, that have a heightened risk for being hospitalized. Adjusting by these factors is a common way to remove differences caused by demographics instead of health systems when comparing different populations. The process of adjusting provides a hypothetical rate that would be observed in a population if, for example, the age distribution of the group were the same as the age distribution of the standard population (9).

In traditional rate calculations, we assume that the events such as hospitalizations (numerator) occur among the population at risk (denominator). When calculating hospitalization rates, the true at-risk population is limited to those who have an ACSC. By using the number of people who report being diagnosed with an ACSC in the denominator, we can calculate a disease prevalence–adjusted rate that should more accurately reflect potentially avoidable hospitalizations.

We assessed the value of incorporating disease prevalence into the denominator in hospitalization-rate calculations. Using California Health Interview Survey (CHIS) data and hospital discharge data, this study examines standard and disease prevalence-adjusted ACSC hospitalization rates for hypertension and congestive heart failure (CHF) among California adults. By comparing a disease prevalence–adjusted rate with standard-rate calculations, we hypothesized that prevalence–adjusted hospitalization rates would highlight areas with higher ACSC burden.

## Methods

We combined population-based data from CHIS and hospitalization data from the California Office of Statewide Health Planning and Development's (OSHPD's) hospital patient discharge files. This study received institutional review board approvals from the University of California, Los Angeles (UCLA) Human Subjects Protection Committee, the California Health and Human Services Agency, and the California OSHPD.

### Data sources

Conducted every other year since 2001, CHIS is a random-digit–dialed telephone survey of California's noninstitutionalized population. On average, there are 45,000 completed CHIS interviews with adults aged 18 years or older per data collection year. CHIS data from 2003, 2005, and 2007 were pooled and weighted to adjust for geographic oversampling and to reflect California population characteristics. ACSC prevalence, population size, and demographic characteristics were obtained at the county level from CHIS data.

The OSHPD hospital patient discharge dataset comprises a record for each inpatient discharged from a licensed acute care hospital in California. We used patient discharge data from OSPHD of respondents aged 18 years or older from 2004 through 2006, which included more than 11 million records. From the OSPHD dataset, we used principal diagnosis, source or type of admission, discharge date, and patient-level characteristics. Because of small population sizes, 19 counties were grouped into 4 county clusters. Both Los Angeles and San Diego counties were subdivided into smaller subcounty areas. Data were managed and analyzed in Microsoft Excel 2007 and SAS version 9.2 (SAS Institute, Inc, Cary, North Carolina).

### Identifying ACSC conditions and hospitalizations

We chose 2 chronic conditions on the basis of AHRQ's PQIs: hypertension (PQI no. 7) and CHF (PQI no. 8). For disease prevalence, we used CHIS data of adults reporting ever being diagnosed with hypertension or CHF by a doctor. Estimates and variances of disease prevalence at the county level were pooled from CHIS 2003, 2005, and 2007. For areas with a small number of events, we imputed the disease prevalence estimate on the basis of similar county size characteristics (eg, population size, disease prevalence, hospitalizations). To identify ACSC hospitalizations, we used principal diagnosis as defined by the International Classification of Diseases, Ninth Revision, Clinical Modification (ICD-9-CM) (Appendix A). The average annual number of hospitalizations between 2004 and 2006 was used to obtain more stable hospitalization rates at the county level from OSHPD data.

### Rate calculations

We calculated crude, disease prevalence–adjusted, age-adjusted, and combined age-and prevalence-adjusted hospitalization rates for hypertension and CHF. Crude rates were calculated with number of hospitalizations in county as the numerator and total population in county as the denominator (9). For disease prevalence–adjusted rates, the total number of people reporting the ACSC in the county served as the denominator. We used the direct method to calculate the age-adjusted hospitalization rate. First, age-specific hospitalization rates were calculated for each age group using the total number of hospitalizations for a given age group as the numerator and  the total number of people in the population for a given age group as the denominator (9,11). Second, age-specific weights were calculated for each age group to capture the frequency of people in the total population for each age group. By summing the product of the age-specific hospitalization rate with the age-specific weights, we obtained the age-adjusted hospitalization rate. To incorporate disease prevalence into this rate, the total number of people reporting the ACSC served as the denominator for the age-specific hospitalization rates. The 95% confidence intervals for each hospitalization rate were also calculated to account for sampling variation (Appendix B).

All rates were aggregated at the California county level and subcounty level for Los Angeles and San Diego counties. Hospitalization rates were expressed per 100,000 people in the population (for the crude and age-adjusted rates) or per 100,000 people reporting the ACSC in the population (for the disease prevalence–adjusted and age- and disease prevalence–adjusted rates). To compare across counties, age-adjusted and age- and disease prevalence-adjusted rates were standardized (ie, process of comparing counties) by using 2000 US Census population data (12). To compare different rate calculations, we ranked counties from lowest (rank of 1) to highest (rank of 55) by their hospitalization rate. Counties were then grouped into quintiles with group 1 representing areas with the lowest hospitalization rates (indicating better access to or quality of primary care) and group 5 representing areas with the highest rates (indicating the worst access to or quality of primary care).

## Results

In this study, we compared hospitalization rates that do and do not incorporate disease prevalence. We first present disease prevalence, average number of hospitalizations per year, and age-specific rates. Next, age-adjusted and combined age- and disease prevalence–adjusted hospitalization rates are discussed. Results are presented separately for each ACSC.

### Hypertension

**Figure 1.**
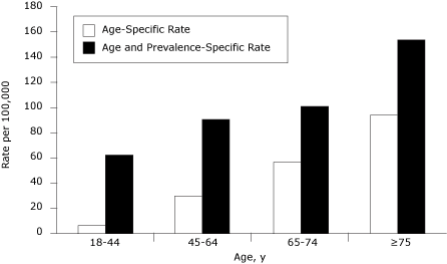
Age-specific and age- and prevalence-specific hospitalization rates for hypertension among California adults. Source: California Health Interview Survey and OSHPD Hospital Patient Discharge Data. Abbreviation: Office of Statewide Health Planning and Development.

The annual prevalence of hypertension was high (24.8%) (Table 1). Despite high prevalence, the average number of hospitalizations was low (6,355 total statewide per year). Older adults showed the highest age-specific hospitalization rate for hypertension at 95 per 100,000 people aged 75 years or older compared with people aged 65 to 74 years (58 per 100,000), 45 to 64 years (30 per 100,000), and 18 to 44 years (7 per 100,000) (Figure 1). After accounting for disease prevalence, the population at risk for hospitalizations became smaller, and thus the age-specific rates per 100,000 people were higher but followed the same age pattern (Figure 1). In comparison with the age-specific rate, the age- and disease prevalence–specific rate among people aged 75 years or older was about 50% higher (154 per 100,000) among those who reported ever being diagnosed with hypertension compared with the total population.

**Table:** 

**Characteristic Age, y**	**Age-Specific Rate**	**Age- and Prevalence-Specific Rate**
18-44	6.5	62.2
45-64	30.4	90.9
65-74	57.7	101.3
≥75	94.5	154.4

**Figure 2.**
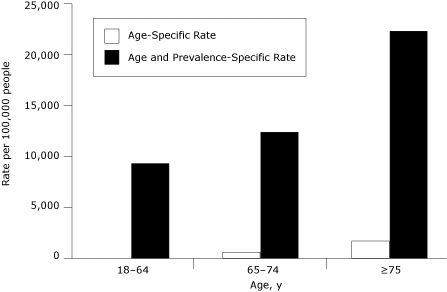
Age-specific and age- and prevalence-specific hospitalization rates for CHF among California adults. Source: California Health Interview Survey and OSHPD Hospital Patient Discharge Data. Abbreviation: OSHPD, Office of Statewide Health Planning and Development.

**Table:** 

**Characteristic Age, y**	**Age-Specific Rate**	**Age- and Prevalence-Specific Rate**
18-64	81.6	9,338.3
65-74	672.5	12,340.5
≥75	1,774.7	22,322.3

There was a low hospitalization rate of hypertension at 24 per 100,000 people (Table 1). After accounting for disease prevalence, the rate was 97 per 100,000 people who reported ever being diagnosed with hypertension. After adjusting for age, the rates were 25 for the age-adjusted rate and 82 for the age- and disease prevalence-adjusted rate. Comparing across county rankings, counties that ranked low for the age-adjusted rate also ranked low for the combined adjusted rate (Table 2). A similar pattern emerged for counties that had high age-adjusted rates and high age- and prevalence-adjusted rates. Only 14 counties differed in group ranking by 1 position.

### Congestive heart failure

The annual prevalence of CHF (1.6%) was much lower than the hypertension prevalence. In contrast, the average number of hospitalizations was higher (65,389 per year) (Table 1). Across age groups, the highest age-specific hospitalization rate appeared among adults aged 75 years or older (1,1,775 per 100,000) followed by adults aged 64 to 74 years (673 per 100,000) and 18 to 64 years (82 per 100,000) (Figure 2). After adjusting for age and disease prevalence, the age-specific rates increased substantially because of the small prevalence of CHF in the total population. Both rates showed a similar pattern in age distribution (Figure 2).

The overall CHF hospitalization rate was 249 per 100,000 (Table 1). After accounting for disease prevalence, the overall rate jumped to 16,773 hospitalizations per 100,000 people who reported ever being diagnosed with CHF. The CHF hospitalization rate increased slightly from the crude rate of 249 to the age-adjusted rate of 270. The combined age- and prevalence-adjusted hospitalization rate was 10,633 per 100,000 people who reported ever being diagnosed with CHF. Accounting for age reduced the CHF hospitalization rate in the combined adjusted rate.

CHF rate comparisons show that the group rankings of 31 counties changed, and among them, 15 counties changed by 2 or more quintiles  (Table 3). Of the 11 counties that ranked the best in the age-adjusted rates, 3 counties changed by at least 2 positions from age-adjusting to the combined age- and prevalence-adjusted rate. Los Angeles SPA 5 ranked in group 1, and Santa Barbara and Santa Clara only ranked in group 2 in the age-adjusted rates; all 3 of them ranked in group 5 in the age-and prevalence-adjusted rates. Most of the counties that ranked the worst for the age-adjusted rates remained ranked in group 4 or group 5 in the combined age- and prevalence-adjusted rates. Stanislaus County and Los Angeles SPA 1-Antelope Valley were the exceptions; they ranked in group 5 in the age-adjusted rate and rose to group 3 in the combined rate.

## Discussion

This study examined the effect of incorporating disease prevalence into the denominator when calculating hospitalization rates for hypertension and CHF. CHF has a low prevalence in the overall population and a high hospitalization rate, and hypertension has a high prevalence in the overall population and a low hospitalization rate. After incorporating disease prevalence into the rate calculation beyond age adjusting, 31 counties were shifted to a new CHF group ranking, compared with only 14 counties that were shifted to a new hypertension group ranking.

Higher ACSC hospitalization rates indicate poor quality, uncoordinated care, or insufficient access to health care (7). Yet, the choice of rate calculation for ACSC hospitalizations can lead to different conclusions about the effectiveness of primary care services. Crude rates measure the overall burden of hospitalizations in a population; age-adjusted rates serve as a relative index of risk that roughly adjusts for age differentials in disease risk of the population (12). These standard rate calculations capture the whole population rather than a population that is truly at risk for hospitalizations and may underestimate the overall burden of preventable hospitalizations. With appropriate disease management and lifestyle modifications, hypertension and congestive heart failure (CHF) are chronic conditions that are largely controllable in outpatient settings. Yet, the complications of CHF are more common and evident than those for hypertension; CHF is the leading cause of hospitalization in California, especially among older adults (8,13).

Little change occurred when accounting for hypertension prevalence in the population at risk for a hospitalization. Counties with high hospitalization rates, regardless of accounting for disease prevalence, continue to be critical areas for improving outpatient care for hypertension. These findings on hypertension refute our hypothesis that adjusting for disease prevalence would highlight areas of higher disease burden. Although overall hospitalization rates are low for hypertension, a 2010 California report showed a dramatic increase in hypertension hospitalization rates from 1999 through 2008; the largest increase occurred from 2006 through 2008 (outside this study period) (8). Future studies should still consider disease prevalence in calculating hypertension hospitalization rates.

Hospitalizations for CHF may be more preventable. After adjusting for disease prevalence, more than half of California counties changed group rankings. Furthermore, counties that reported lower group rankings by standard calculations switched to higher group rankings when adjusting for CHF prevalence. Areas such as SPA 5-West Area in Los Angeles County, Santa Barbara County, and Santa Clara County all shifted from low to high hospitalization rates. Although these are more affluent areas with high incomes and low poverty levels, the low rate of hospitalizations and high rate when adjusted for CHF prevalence may point to a higher tendency to hospitalize people with CHF. Factors such as low disease prevalence and large number of hospital beds may also fuel these hospitalization rates and thus create a hospital supply-induced care rather than a need-induced care. Although we cannot differentiate between supply-induced and need-induced care, these rate calculations are based on a patient's zip code of residence and not on referrals into an area with better hospitals. Further research is needed to explain the higher prevalence-adjusted hospitalization rates in these areas to help reduce preventable CHF hospitalizations.

This study has several limitations. First, disease prevalence is measured from a population-based survey, whereas hospitalizations are based on an administrative census count. The underdiagnosis of chronic conditions is well established from population-based surveys (14,15), and so CHIS's self-reported awareness of diagnosed hypertension and CHF may underestimate the true prevalence. The rates we calculate will therefore be somewhat inflated, but we do not expect any significant bias by county. Previous literature has indicated data limitations for hospitalization data, including poor quality control and overestimation of disease trends (16-18). California's OSHPD regularly conducts a series of audits to ensure validity of hospitalization data. If data reports do not meet error tolerance levels of less than 0.1%, OSHPD sends the data back to the hospital to be corrected (19). To address the potential overestimation by OSHPD, we limited hospital records to principal diagnosis of hypertension and CHF. Although we weighted the population-based CHIS data and examine variance of the hospitalization rates,  sampling biases may limit the accuracy of the findings.

Second, hospitalization rates have been shown to vary widely by sex and race/ethnicity (20-25). Because of low ACSC prevalence in some counties, it was difficult to obtain stable estimates if we stratified by more than 1 demographic characteristic. We chose to adjust by age because hospitalizations vary most widely between age groups, as evident from the age-specific rates. Third, categories for CHF generated small sample sizes that may lead to high variance when incorporating CHF disease prevalence into the denominator. This issue affects rate calculations most when we stratify by age groups at the county level. We attempted to reduce the variation by using pooled CHIS data across 3 years and imputing age-specific disease prevalence where sample sizes are too small to report.

The results from this study show that disease prevalence should be incorporated into ACSC research. Previous studies have included disease prevalence as a control variable in statistical analysis rather than incorporating disease prevalence into the hospitalization rate (23,26-28). By incorporating prevalence into hospitalization rate calculations, we can assess the true population at risk who have the disease instead of just the population at large who reside in the area. Taking into account disease prevalence can highlight areas with higher burden of chronic conditions on the health care system (both inpatient and outpatient). Because of rising health care costs and the anticipated health care reform changes to the health care system, our current tools for evaluating health services should be assessed. Our proposed method of calculation builds on existing methods and offers a relatively simple alternative by accounting for people who are truly at risk for ACSC hospitalization. By using more accurate measures, public health officials and health care providers can develop more effective community health interventions and improve outpatient care for ACSC.

## Figures and Tables

**Table 1:** ACSC Prevalence and Hospitalizations, California Adults Aged 18 Years or Older^a^

**Characteristic**	Congestive Heart Failure, N (95% CI)	Hypertension, N (95% CI)
**Total prevalence**	389,839 (375,400-404,278)	6,527,573 (6,464,510-6,590,636)
**Percentage of population**	1.6%	24.8%
**Prevalence by age, y**		
18-44	197,000 (178,494-215,506)^b^	1,511,000 (1,456,354-1,565,646)
45-64		2,730,000 (2,676,325-2,783,675)
65-74	107,000 (97,418-116,582)	1,123,000 (1,089,103-1,156,896)
≥75	151,000 (140,096-161,904)	1,164,000 (1,130,777-1,197,223)
**Average hospitalization per year**	65,389	6,355
**Hospitalizations by age, y**		
18-44	18,379^b^	940
45-64		2,480
65-74	13,256	1,138
≥75	33,755	1,797

	**Rate (95% CI)**	**Rate (95% CI)**

**Crude hospitalization rate^c^**	248.8 (248.75-248.77)	24.1 (24.17-24.18)
**Prevalence-adjusted hospitalization rate^d^**	16,773.3 (16,771.33-16,775.34)	97.4 (97.35-97.36)
**Age-adjusted hospitalization rate^c^**	270.2 (266.0-274.4)	25.1 (24.4-25.8)
**Age- and prevalence-adjusted hospitalization rate^d^**	10,633.0 (9,875.7-11,390.4)	81.5 (78.6-84.3)

Abbreviations: ACSC, ambulatory care–sensitive conditions; OSHPD, Office of Statewide Health Planning and Development; CI, confidence interval.

a Data were pooled from the California Health Interview Survey for 2003, 2005, and 2007 and averaged from OSHPD discharge files for 2004, 2005, and 2006.

b Age groups 18-44 y and 45-64 y are combined for this rate because of the small prevalence of congestive heart failure in the population.

c Rate per 100,000 people in the population.

d Rate per 100,000 people reporting ACSC in the population.

**Table 2:** Age-Adjusted and Age- and Prevalence-Adjusted Hospitalization Rates for Hypertension, California Adults 18 Years or Older^a^^,^ b

**County Cluster/County/Subcounty**	Hypertension			

	Age-Adjusted Rate		Age- and Prevalence**–**Adjusted Rate	

	Rate (95% CI)	Group	Rate (95% CI)	Group
**California**	25.1 (24.4-25.8)	NA	81.5 (78.6-84.3)	NA
Alameda	21.2 (18.3-24.2)	4	73.2 (59.2-87.2)	4
Butte	25.0 (17.6-32.5)	4	76.0 (45.5-106.6)	4
Contra Costa	15.3 (12.3-18.2)	2	45.1 (34.2-56.1)	2
Del Norte, Siskiyou, Lassen, Trinity, Modoc, Plumas, Sierra	10.0 (4.9-15.2)	1	34.5^c^ (12.3-56.7)	1
El Dorado	19.9 (12.1-27.6)	3	82.6^c^ (34.4-130.8)	4
Fresno	17.9 (14.1-21.7)	3	58.0 (43.1-72.8)	3
Humboldt	8.6^c^ (2.7-14.5)	1	39.3^c^ (3.7-74.9)	1
Imperial	28.1 (17.6-38.6)	4	86.0 (46.4-125.7)	4
Kern	25.0 (20.1-30.0)	4	70.8 (53.7-87.9)	4
Kings	33.0 (19.0-47.0)	5	90.8 (47.0-134.6)	4
Lake	10.7 (3.4-18.0)	1	35.0^c^ (4.2-65.8)	1
Los Angeles SPA 1 – Antelope Valley	43.0 (33.1-52.9)	5	139.8 (100.3-179.3)	5
Los Angeles SPA 2 – San Fernando	32.4 (29.1-35.7)	5	100.6 (87.1-114.1)	5
Los Angeles SPA 3 – San Gabriel Valley	29.9 (26.6-33.2)	4	94.3 (79.5-109.0)	5
Los Angeles SPA 4 – Metro	71.8 (64.1-79.4)	5	219.9 (189.3-250.4)	5
Los Angeles SPA 5 – West Area	13.5 (10.4-16.7)	2	45.7 (30.5-60.9)	2
Los Angeles SPA 6 – South	75.8 (66.0-85.6)	5	201.6 (169.0-234.1)	5
Los Angeles SPA 7 – East Area	35.3 (30.7-39.9)	5	111.9^c^ (93.1-130.7)	5
Los Angeles SPA 8 – South Bay	30.9 (27.3-34.5)	5	98.5^c^ (83.5-113.5)	5
Madera	15.3 (7.4-23.2)	2	42.3^c^ (15.1-69.6)	1
Marin	11.1 (6.5-15.7)	1	48.5 (13.6-83.4)	2
Mendocino	6.4^c^ (0.5-12.4)	1	18.7 (0.0-42.4)	1
Merced	18.5 (11.2-25.8)	3	58.0^c^ (29.6-86.3)	3
Monterey	20.5 (15.1-26.0)	4	77.6^c^ (48.2-107.0)	4
Napa	13.1^c^ (6.1-20.2)	2	43.5 (10.2-76.8)	2
Nevada	17.4^c^ (9.1-25.7)	3	78.2^c^ (7.0-149.4)	4
Orange	25.2 (22.7-27.7)	4	85.6 (73.3-97.8)	4
Placer	10.9 (6.7-15.1)	1	37.8 (17.6-58.1)	1
Riverside	27.3 (24.3-30.3)	4	88.2^c^ (75.0-101.4)	4
Sacramento	16.7 (14.0-19.4)	3	62.7 (47.1-78.3)	3
San Benito	35.4^c^ (14.4-56.3)	5	95.4 (27.4-163.4)	5
San Bernardino	40.1 (35.8-44.3)	5	117.4 (102.3-132.5)	5
San Diego Region 1--North Coastal	13.4 (9.5-17.3)	2	40.2 (25.7-54.7)	1
San Diego Region 2--North Central	14.7 (10.9-18.6)	2	49.2 (32.8-65.6)	2
San Diego Region 3--Central	34.0 (26.1-41.9)	5	98.2 (70.2-126.3)	5
San Diego Region 4--South	20.0 (14.2-25.7)	4	65.0 (41.0-89.1)	3
San Diego Region 5--East	14.8 (10.6-19.0)	2	50.5^c^ (31.1-69.9)	2
San Diego Region 6--North Inland	8.3 (5.2-11.4)	1	28.4 (15.5-41.3)	1
San Francisco	16.9 (13.6-20.2)	3	63.8 (46.6-81.0)	3
San Joaquin	34.7 (28.5-41.0)	5	93.4 (73.9-112.9)	5
San Luis Obispo	5.7 (2.5-8.9)	1	28.2 (3.0-53.3)	1
San Mateo	15.5 (11.9-19.1)	2	43.2 (29.9-56.5)	2
Santa Barbara	11.7 (7.8-15.7)	1	43.3 (23.2-63.5)	2
Santa Clara	14.2 (12.0-16.5)	2	49.9 (39.5-60.2)	2
Santa Cruz	16.0 (9.8-22.2)	2	56.5 (27.5-85.5)	3
Shasta	17.6 (10.8-24.3)	3	56.6 (28.5-84.7)	3
Solano	17.3 (12.2-22.3)	3	51.5^c^ (33.2-69.8)	2
Sonoma	7.8 (4.9-10.7)	1	30.1 (13.6-46.6)	1
Stanislaus	19.6 (14.5-24.7)	3	56.4^c^ (39.8-73.1)	3
Sutter, Yuba	24.4 (14.9-33.9)	4	68.9 (36.4-101.5)	4
Tehama, Glenn, Colusa	17.1 (8.2-26.0)	3	57.9^c^ (20.2-95.6)	3
Tulare	17.5 (12.1-22.9)	3	51.5 (33.1-70.0)	2
Tuolumne, Calaveras, Amador, Inyo, Mariposa, Mono, Alpine	14.3 (8.3-20.3)	2	55.7 (20.8-90.7)	3
Ventura	20.4 (16.4-24.4)	4	65.9 (49.0-82.8)	3
Yolo	9.5 (3.9-15.1)	1	38.5 (12.9-64.2)	1

Abbreviations: CHIS, California Health Interview Survey; OSHPD, Office of Statewide Health Planning and Development; CI, confidence interval; NA, not applicable.

a Rates are per 100,000 people; Group 1 = lowest rate, Group 5 = highest rate.

b Data were pooled from the California Health Interview Survey for 2003, 2005, and 2007 and averaged from OSHPD discharge files for 2004, 2005, and 2006.

c Rates are unstable estimates with a coefficient of variance of more than 30%.

**Table 3:** Age-Adjusted and Age- and Prevalence-Adjusted Hospitalization Rates for Congestive Heart Failure, California Adults Aged 18 Years or Older^a^^,^ b

**County Cluster/County/Subcounty**	Congestive Heart Failure			

	Age-Adjusted Rate		Age- and Disease Prevalence-Adjusted Rate	

	Rate (95% CI)	Group	Rate (95% CI)	Group
**CALIFORNIA**	270.2 (266.0-274.4)	NA	10,633.0 (9,875.7-11,390.4)	NA
Alameda	302.2 (279.5-324.9)	4	11,322.5 (7,690.6-14,954.4)	4
Butte	271.6 (238.9-304.2)	4	9,497.9 (4,102.5-14,893.3)	3
Contra Costa	236.6 (215.6-257.6)	3	11,103.2 (6,375.9-15,830.5)	3
Del Norte, Siskiyou, Lassen, Trinity, Modoc, Plumas, Sierra	174.4 (149.4-199.5)	1	6,524.5^c^ (1,776.5-11,272.6)	1
El Dorado	203.5 (168.7-238.3)	2	8,124.1 (4,445.2-11,802.9)	2
Fresno	325.4 (286.3-364.5)	5	24,644.1^c^ (7,657.9-41,630.2)	5
Humboldt	224.7 (184.8-264.6)	2	8,367.9 (4,707.3-12,028.4)	2
Imperial	315.6 (264.7-366.5)	4	11,415.4 (5,413.9-17,417.0)	4
Kern	316.0 (277.4-354.6)	4	8,233.9^c^ (2,509.6-13,958.2)	2
Kings	305.1 (247.4-362.9)	4	13,014.2 (7,297.9-18,730.5)	4
Lake	188.9 (153.9-223.9)	1	7,836.9 (3,340.8-12,333.0)	1
Los Angeles SPA 1 – Antelope Valley	399.8 (346.4-453.1)	5	11,294.3 (5,223.8-17,364.8)	3
Los Angeles SPA 2 – San Fernando	280.1 (259.0-301.3)	4	11,548.7 (7,947.7-15,149.8)	4
Los Angeles SPA 3 – San Gabriel Valley	285.9 (266.3-305.5)	4	12,520.9 (8,780.9-16,260.8)	4
Los Angeles SPA 4 – Metro	392.5 (356.9-428.1)	5	15,643.3 (10,340.2-20,946.4)	5
Los Angeles SPA 5 – West Area	159.3 (141.8-176.7)	1	17,293.6^c^ (2,270.7-32,316.5)	5
Los Angeles SPA 6 – South	539.1 (483.7-594.5)	5	17,756.4 (8,623.5-26,889.3)	5
Los Angeles SPA 7 – East Area	290.8 (265.0-316.7)	4	13,957.8 (7,276.6-20,639.0)	4
Los Angeles SPA 8 – South Bay	259.0 (240.3-277.8)	3	10,761.9 (6,133.3-15,390.5)	3
Madera	260.7 (219.0-302.5)	3	7,813.2 (4,693.9-10,932.5)	1
Marin	181.6 (156.2-207.1)	1	7,872.5 (4,773.2-10,971.8)	1
Mendocino	221.2 (177.1-265.3)	2	9,182.2 (4,078.7-14,285.7)	2
Merced	326.9 (279.2-374.7)	5	12,081.6 (6,226.1-17,937.1)	4
Monterey	229.6 (198.3-260.9)	3	6,198.8 (4,213.8-8,183.8)	1
Napa	216.7 (181.5-252.0)	2	7,245.5 (3,891.1-10,599.9)	1
Nevada	159.2 (130.1-188.3)	1	6,615.4^c^ (1,968.3-11,262.4)	1
Orange	249.2 (232.6-265.9)	3	10,249.1 (7,488.3-13,009.9)	3
Placer	196.3 (169.9-222.7)	1	10,179.4^c^ (553.0-19,805.7)	3
Riverside	258.3 (242.4-274.3)	3	7,878.7 (5,762.3-9,995.2)	2
Sacramento	276.6 (256.3-296.9)	4	9,406.0 (6,726.6-12,085.4)	3
San Benito	252.3 (175.2-329.3)	3	16,730.9^c^ (0.0-34,669.1)	5
San Bernardino	344.4 (318.2-370.5)	5	12,775.2 (9,302.0-16,248.3)	4
San Diego Region 1 – North Coastal	191.8 (166.8-216.7)	1	9,031.6 (3,106.7-14,956.5)	2
San Diego Region 2 — North Central	189.2 (161.1-217.3)	1	10,827.0 (4,899.0-16,754.9)	3
San Diego Region 3 — Central	419.9 (347.5-492.3)	5	14,329.7 (8,129.2-20,530.1)	4
San Diego Region 4 — South	357.0 (295.4-418.5)	5	12,834.5 (6,621.3-19,047.6)	4
San Diego Region 5 — East	198.7 (171.3-226.1)	2	3,742.7 (2,623.8-4,861.6)	1
San Diego Region 6 — North Inland	186.5 (159.7-213.2)	1	7,352.9^c^ (2,524.6-12,181.1)	1
San Francisco	233.1 (207.2-258.9)	3	21,102.1^c^ (1,439.7-40,764.6)	5
San Joaquin	366.2 (323.2-409.2)	5	25,314.9 (14,549.8-36,080.1)	5
San Luis Obispo	167.2 (142.5-191.9)	1	8,056.2 (3,734.7-12,377.6)	2
San Mateo	203.9 (178.7-229.1)	2	12,556.0^c^ (4,385.9-20,726.0)	4
Santa Barbara	200.3 (172.8-227.8)	2	16,261.1^c^ (0.0-33,121.7)	5
Santa Clara	208.3 (190.1-226.5)	2	16,988.8 (10,321.0-23,656.6)	5
Santa Cruz	270.5 (226.6-314.4)	3	30,677.6^c^ (0.0-73,032.0)	5
Shasta	240.7 (210.3-271.1)	3	11,044.1 (5,026.7-17,061.5)	3
Solano	298.0 (257.8-338.3)	4	8,132.8 (6,096.5-10,169.0)	2
Sonoma	197.3 (171.8-222.9)	1	8,153.6 (4,636.3-11,670.8)	2
Stanislaus	337.0 (295.6-378.3)	5	9,800.9 (5,678.4-13,923.4)	3
Sutter, Yuba	318.9 (274.6-363.2)	5	16,974.0 (9,275.1-24,673.0)	5
Tehama, Glenn, Colusa	258.4 (215.3-301.5)	3	8,172.2 (3,872.2-12,472.2)	2
Tulare	313.9 (273.6-354.2)	4	10,150.5 (5,832.6-14,468.4)	3
Tuolumne, Calaveras, Amador, Inyo, Mariposa, Mono, Alpine	209.2 (180.4-237.9)	2	5,949.4 (2,827.0-9,071.7)	1
Ventura	222.9 (194.3-251.6)	2	7,906.9^c^ (2,494.9-13,318.8)	2
Yolo	221.7 (182.5-260.9)	2	6,356.6 (3,290.6-9,422.6)	1

Abbreviations: CHIS; California Health Interview Survey; OSHPD, Office of Statewide Health Planning and Development; CI, confidence interval.

a Rates are per 100,000 individuals; Group 1 = lowest rate, Group 5 = highest rate.

b Data were pooled from the California Health Interview Survey for 2003, 2005, and 2007 and averaged from OSHPD discharge files for 2004, 2005, and 2006.

c Rates are unstable estimates with a coefficient of variance of more than 30%.

## Appendices

### Appendix A. Measures for Ambulatory Care–Sensitive Conditions from OSHPD and CHIS Data

**Table:**

**ACS Condition**	**Hospitalizations^a^ by ICD-9-CM Codes** (OSHPD Principal Diagnosis)	**Disease Prevalence **(CHIS Self-Report)
**Congestive heart failure** (PQI #8)	428, 402.01, 402.11, 402.91, 518.4 Age groups: 18-64, 65-74, and ≥75 y	Adults who reported ever having been diagnosed with congestive heart failure by a doctor.
**Hypertension** (PQI #7)	401.0, 401.9, 402.00, 402.1, 402.9 Age groups: 18-44, 45-64, 65-74, and ≥75 y	Adults who reported ever having been diagnosed with high blood pressure or hypertension by a doctor.

Abbreviations: OSHPD, Office of Statewide Health Planning and Development; CHIS, California Health Interview Survey; ICD-9-CM, *International Classification of Diseases, Ninth Revision, Clinical Modification.*

a California hospitals include general acute care hospitals, acute psychiatric hospitals, chemical dependency recovery hospitals, and psychiatric health facilities. Excludes transfer from a hospital (different facility), a skilled nursing facility or intermediate care facility, or another health care facility; and MDC 14 (pregnancy). Only 1 hospital record was used for patients who had multiple hospital admissions for the same principle diagnosis per year.

### Appendix B. Methods for Calculating Hospitalization Rates and Data Sources

**Table:**

**Standard Methods**
**1. Crude Hospitalization Rate**
Total # Hospitalizations in County
____________________________________________
Total # People in County
Numerator: OSHPD data 2004-2006
Denominator: CHIS data, 2005 population estimates
**Variance =**
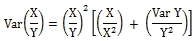

**2. Age-Adjusted Hospitalization Rate**
Σ[Age-Specific Rates _(Age Group 1)_ X (Standard Population _(Age Group 1)_]
_____________________________________________________________
Total # People in Standard Population
Age-Specific Rates =
[Total # Hospitalizations in County _(Age Group 1)_]
____________________________________________
[Total # People inCounty _(Age Group 1)_]
Age- and Prevalence-Specific Rate Numerator: OSHPD data 2004-2006
Age- and Prevalence-Specific Rate Denominator: CHIS data (2003, 2005, 2007) disease prevalence estimates
**Variance** = Sum of the age-specific rate variances
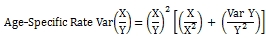

**Methods That Incorporate Disease Prevalence**

**3. Prevalence-Adjusted Hospitalization Rate**
Total # Hospitalizations in County
____________________________________________
Total # People Reporting ACS Condition in County
Numerator: OSHPD data, 2004-2006
Denominator: CHIS pooled data (2003, 2005, 2007) disease prevalence estimates
**Variance =**
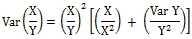

**4. Age- and Prevalence-Adjusted Hospitalization Rate**
Σ[Age- and Prevalence-Specific Rates _(Age Group 1)_ X (Standard Population _(Age Group 1)_]
_________________________________________________________________
Total # People in Standard Population
Age- and Prevalence-Specific Rates =
Total # Hospitalizations in County _(Age Group 1)_
_________________________________________________
[Total # People Reporting ACS Condition in County_(Age Group 1)_]
Standard Population: US Census 2000 population
Age- and Prevalence-Specific Rate Numerator: OSHPD Data 2004-2006
Age- and Prevalence-Specific Rate Denominator: CHIS Data (2003, 2005, 2007) Disease Prevalence Estimates
**Variance** = Sum of the age-specific rate variances
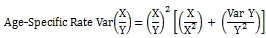

Abbreviations: OSHPD, Office of Statewide Health Planning and Development; CHIS, California Health Interview Survey; ACS, ambulatory care sensitive.
