# Erratum, Vol. 8: Issue 5

**Published:** 2011-10-15

In the article "A Common Denominator: Calculating Hospitalization Rates for Ambulatory Care–Sensitive Conditions in California," the citation for reference 12 was incomplete. The correction was made to our website on October 17, 2011, and appears online at http://www.cdc.gov/pcd/issues/2011/sep/11_0013.htm. We regret any confusion or inconvenience this error may have caused.

